# Particular gene order and complete mitochondrial genome of *Ariosoma meeki* (Congridae, *Ariosoma*)

**DOI:** 10.1080/23802359.2019.1681914

**Published:** 2019-10-25

**Authors:** Qing Chu, Jiong Chen, Tianjun Xu

**Affiliations:** a Laboratory of Biochemistry and Molecular Biology, School of Marine Sciences, Ningbo University, Ningbo, China;; b Laboratory of Marine Biology and Biotechnology, Qingdao National Laboratory for Marine Science and Technology, Qingdao, China;; c Laboratory of Fish Molecular Immunology, College of Fisheries and Life Science, Shanghai Ocean University, Shanghai, China

**Keywords:** Congridae, *Ariosoma meeki*, mitochondrial genome, phylogenetic analysis

## Abstract

*Ariosoma meeki* (*A. meeki*) is a demersal and carnivorous fish species belongs to the family Congridae. Some wild populations of *A. meeki* are in danger because of the overfishing and environmental pollution. In order to better understand the germplasm resources, the complete mitochondrial genome of *A. meeki* was firstly determined in this study. The complete mitochondrial genome is 16,404 nucleotides, comprising 12 protein-coding genes, 2 ribosomal RNA genes, 20 tRNA genes, and 2 main non-coding regions, but ND6 and two tRNA genes were not found in the *A. meeki* mitogenome. The *A. meeki* mitogenome was currently the first member of *Ariosoma* genus with gene and tRNA deletion. In addition, phylogenetic analysis result demonstrated that *A. meeki* and *A. shiroanago* were clustered in a clade and formed a sister relationship.

Congridae was found worldwide in tropical, sub-tropical and temperate latitudes and included three subfamily (Heterocngrinae, Bathymyrinae and Congrinae). *Ariosoma meeki* (*A. meeki*), one species of *Ariosoma* genus belongs to Congrinae subfamily, is widespread on coastal and continental shelf areas. Congrinae is at a risk of extinction due to overfishing, the declines in its abundance, and narrow range of its distribution. In order to better understand the germplasm resources, the complete mitochondrial genome of *A. meeki* was firstly determined in this study.

We sequenced the complete mitogenome of the *A. meeki* which was collected from the East China Sea (29.9′N, 122.2′E) and was kept at the Museum of Marine Biology at Zhejiang Ocean University (Index number: MMB-2014-0116). The mitogenome of *A. meeki* was a closed double-stranded circular molecule of 16,404 nucleotides (GenBank accession no.: KX641476) and consisted of 12 protein genes, 2 rRNA genes, 20 tRNA genes, 2 main non-coding regions (the control region and the origin of the light strand replication). Meanwhile, the gene order of most genes and gene coding strand of *A. meeki* mitogenome conformed to the vertebrate consensus (Sun and Xu 2018). However, the ND6 gene, tRNA^Glu^ and tRNA^Pro^ were not found in the mitogenome of *A. meeki*, which is different with other species. The deletion of ND6 gene and two tRNA is found only in the *A. meeki*, we speculated that the mitogenome from *Ariosoma* genus may appear the deletion. In addition, the overall base composition was T 26.5%, C 25.3%, A 28.5%, and G 19.8%. The A + T content was higher than G + C content, which is similar to other fishes (Cheng et al. 2012). An anti-G bias was ascertained in the third position of protein-coding genes, which is similar to other vertebrate mitogenomes (Cheng et al. 2011; Jin et al. 2012). The 12 protein-coding genes were encoded on heavy strand and the ND6 gene was not found in *A. meeki* mitogenome. All the protein-coding genes started with ATG, except for COI which is used GTG as the initiation codon. The stop codon of five protein-coding genes (COII, ATPase8, COIII, ND4L, and Cytb) was TAA and three protein-coding genes (ND1, ND2, and ND3) were TAG, while COI ended with AGA. The remaining protein-coding genes (ATPase6 and ND4) had incomplete stop codons, either TA– or T––, which is common among vertebrate mitochondrial protein-coding genes (Ojala et al. 1981). And, these incomplete termination codons were presumably completed as TAA via posttranscriptional polyadenylation (Ojala et al. 1981). The two ribosomal RNA genes (12S and 16S) were located between tRNA^Phe^ and tRNA^Leu^ (UUR) and were separated by tRNA^Val^ gene. The 20 tRNA genes, ranging from 62 to 74 nucleotides, could fold into a typical cloverleaf structure except for tRNA^Ser^ (AGY) which lacked a dihydrouridine arm. As in most vertebrates, the origin of L-strand replication from *A. meeki* was found between tRNA^Asn^ and tRNA^Cys^, which could fold into a stem-loop secondary structure.

Phylogenetic analysis result based on the complete mitogenome sequences demonstrated that *A. meeki* and *A. shiroanago* were clustered in a clade and formed a sister relationship (Figure 1). We expect that the researches will facilitate further investigations on the taxonomic resolution and phylogenetic relationships of Congridae.

**Figure 1.**
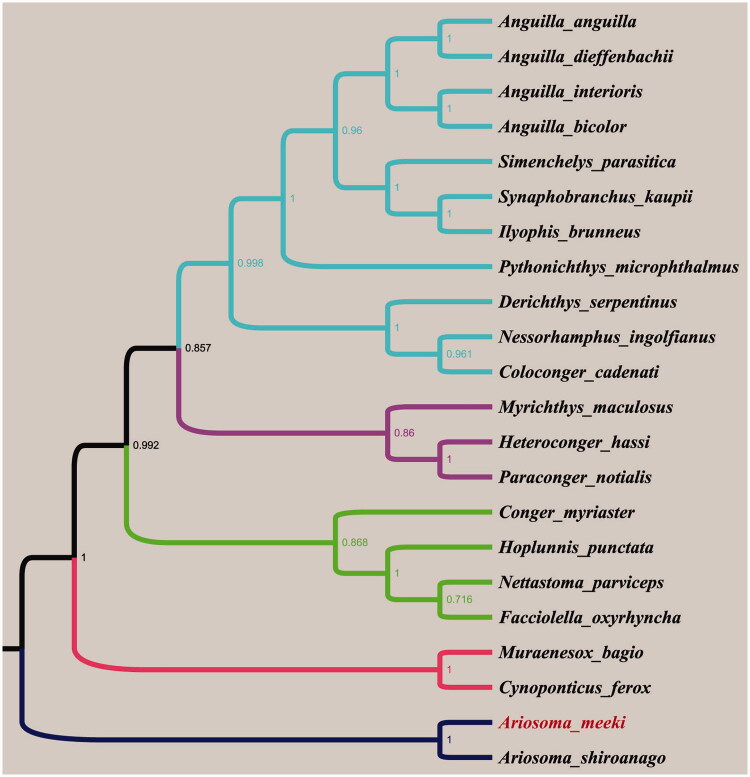
Phylogenetic tree based on the complete mitochondrial genome sequences was constructed using Bayesian method. The numbers in topologies represent Bayesian posterior probability values. The other mitochondrial genome sequences used in phylogenetic analyses were derived from GenBank, accession no.: *Anguilla anguilla* AP007233.1, *Anguilla dieffenbachii* AP007240.1, *Anguilla interioris* AP007241.1, *Anguilla bicolour* AP007237.3, *Ariosoma shiroanago* AP010861.1, *Coloconger cadenati* AP010863.1, *Conger myriaster* AB038381.2, *Cynoponticus ferox* AP010853.1, *Derichthys serpentinus* AP010851.1, *Facciolella oxyrhyncha* AP010866.1, *Heteroconger hassi* AP010859.1, *Hoplunnis punctata* AP010865.1, *Ilyophis brunneus* AP010848.1, *Muraenesox bagio* AP010852.1, *Myrichthys maculosus* AP010862.1, *Nessorhamphus ingolfianus* AP010850.1, *Nettastoma parviceps* AP010864.1, *Paraconger notialis* AP010860.1, *Pythonichthys microphthalmus* AP010842.1, *Simenchelys parasitica* AP010849.1, *Synaphobranchus kaupii* AP002977.2.
